# Diurnal variability of dust transport controlled by mountain terrain and thermal winds

**DOI:** 10.1038/s41598-026-47941-5

**Published:** 2026-04-09

**Authors:** Ramin Ahmadi, Omid Alizadeh, Samaneh Sabetghadam

**Affiliations:** 1 Institute of Geophysics, University of Tehran, Tehran, Iran; 2 Geography Department, Humboldt-Universität zu Berlin, Berlin, Germany

**Keywords:** Dust emission, Dust transport, Dust concentration, Diurnal variability, Planetary boundary layer (PBL) height, Thermally-driven winds, Climate sciences, Environmental sciences

## Abstract

Semi-arid mountain-basin regions, such as Tehran Province in Iran, often experience significant dust pollution in summer, yet the factors controlling its diurnal variability remain poorly understood. This study examines the daily patterns of summer dust events in Tehran, combining hourly MERRA-2 (1980–2023) and ERA5 (2023) reanalysis data with surface PM2.5 and PM10 measurements from six air-quality monitoring stations collected during summer 2023. Reanalysis results show that dust concentrations near the surface are strongly associated with local wind patterns, atmospheric mixing, and transport from surrounding arid plains. Dust emissions peak in the morning due to strong southeasterly winds, which carry dust from desert sources toward populated areas by upslope flows. Surface observations confirm these patterns: southern stations exhibit pronounced nocturnal PM2.5 and PM10 peaks, while central and northern stations display daytime maxima, consistent with northward advection of dust-laden air. MERRA-2 dust surface concentrations exhibit distinct spatial behavior. In southern Tehran, concentrations peak at night and remain elevated into the early morning, followed by a marked afternoon decline as boundary layer height increases. In contrast, central stations display dominant late-morning maxima before midday. In northern areas, the maximum occurs later and the afternoon decrease is less pronounced due to sustained northward dust transport that partially offsets vertical dilution. At night, cooler mountain breezes reduce local dust concentrations, although levels remain elevated over the desert plains. The observed diurnal cycle results from the interplay of thermal mountain breezes, shifting wind directions, and boundary layer dynamics, which together govern dust emission, transport, and dilution. These findings highlight how mountainous terrain and daily temperature changes shape dust pollution patterns, providing insights for air quality management in semi-arid mountain-basin regions worldwide.

## Introduction

Dust storms in arid and semi-arid regions significantly affect air quality, human health, and climate, with complex terrain shaping their transport and dispersion. Tehran Province, situated at the southern edge of the Alborz Mountains, exemplifies this complexity. Diurnal mountain-basin winds drive distinctive upslope and downslope flows, critically influencing dust dynamics in the region.

During daytime, southeasterly upslope winds transport dust from the arid plains toward the Alborz Mountains, while at night, northeasterly downslope winds reverse this flow. These surface winds are coupled with return flows at higher altitudes, creating complex vertical wind profiles that vary over the diurnal cycle^1,2^. Furthermore, topography strongly modulates the planetary boundary layer (PBL) height, which exhibits pronounced diurnal changes impacting pollutant dispersion^3^.

Iran’s predominantly arid and semi-arid climate^4^, along with extensive dust sources such as the Kavir Desert^5^, makes dust storms frequent across Tehran Province year-round. The southeastern plains, including the Kavir and Varamin regions, serve as key dust sources transported by southeasterly winds into Tehran^6^. Recent studies link dust emission variability primarily to near-surface wind speeds and soil moisture deficits, especially during summer when dust activity peaks due to strengthened winds and increased soil desiccation^5^. Anthropogenic land-use changes and recurrent droughts have further exacerbated dust event frequency and intensity in the region^7–9^.

In Tehran city, dust concentrations exhibit strong seasonal and diurnal variations. Summer dust storms dominate aerosol loading, significantly increasing fine (PM2.5) and coarse (PM10) particulate matter levels^10,11^. High concentrations of dust near the surface correlate inversely with PBL height, which reaches a maximum in summer afternoons, suggesting a critical role of PBL dynamics in dust dispersion^12^.

Diurnal cycles of pollutants in Tehran reflect the interplay between thermally-driven mountain-valley winds and PBL structure. Nocturnal downslope flows enhance pollutant accumulation near the surface by reducing atmospheric mixing and PBL height, leading to peak concentrations of fine particles in early morning hours^10,13^. Despite extensive studies on urban pollutants, the diurnal variability of dust aerosols over Tehran Province, and its meteorological controls, remains insufficiently characterized.

Understanding diurnal dust dynamics is essential, given their impacts on air quality, visibility, and public health risks such as respiratory and cardiovascular diseases^14,15^. Unlike localized urban emissions, dust transport in Tehran Province is strongly influenced by advection from external sources, complicating predictions and mitigation.

This study investigates the diurnal characteristics of dust-related variables in Tehran Province during summer, focusing on the influence of complex topography, mountain-basin circulations, and PBL dynamics. Our objectives are to (1) characterize diurnal dust concentration and flux patterns, (2) examine interrelationships among dust and meteorological variables, and (3) identify dominant factors driving these variations. By enhancing understanding of dust emission and transport processes in mountainous semi-arid environments, this work aims to improve forecasting and inform strategies to mitigate dust impacts in Tehran and similar regions.

## Data description and methodology

### Data

In this study, we utilized hourly data from the Modern-Era Retrospective analysis for Research and Applications, version 2 (MERRA-2), with a horizontal resolution of 0.625° × 0.5°, covering the period from 1980 to 2023. The data was used to analyze diurnal variations in dust-related variables during the summer months (June, July, and August). MERRA-2, developed by NASA’s Global Modeling and Assimilation Office (GMAO), is the latest reanalysis product, incorporating new observational data that were unavailable in its predecessor, MERRA. It also uses updated versions of the GEOS model^16^.

The quantities taken from MERRA-2 include dust emission flux, dust surface mass concentration (referred to as dust surface concentration), dust column u-wind and v-wind mass flux (also referred to as dust horizontal flux), PBL height, and wind data at 10- and 50-meter heights.

To analyze diurnal variations in vertical wind structure and characterize three-dimensional thermally driven circulations, we also used hourly data for summer 2023 from the European Centre for Medium-Range Weather Forecasts (ECMWF) Reanalysis 5th Generation (ERA5), which has a horizontal resolution of 0.25° × 0.25°^17^. Additionally, we utilized geopotential height, omega (vertical velocity in pressure coordinates), and zonal and meridional wind components at various pressure levels from ERA5. The higher resolution of ERA5, compared to MERRA-2, allows for more precise capture of detailed wind patterns in localized areas such as Tehran Province.

Hourly PM2.5 and PM10 measurements were also used to provide observational support for the simulated diurnal variability of dust-related aerosols over Tehran. Although PM2.5 and PM10 include both dust and other aerosol components, no direct surface dust measurements are available in Tehran to enable a direct validation of the MERRA-2 dust products.

The dust mass flux in the vertical direction at the Earth’s surface is referred to as dust emission flux^18^. In this study, dust emission flux was used to identify dust sources and quantify the amount of dust lifted per square meter per hour. To assess horizontal dust transport, we utilized the dust column u-wind and v-wind mass flux components, which represent the vertically integrated horizontal transport of dust mass in the east-west (u-wind) and north-south (v-wind) directions, respectively. These fluxes are computed over the entire atmospheric column (from the surface to the model top) based on mass conservation principles, and are not restricted to the PBL^19^. In the MERRA-2 dataset, the PBL is determined based on the total eddy diffusion coefficient of heat^20^.

In MERRA-2, dust emission is parameterized using wind-dependent formulations for five particle size classes defined by particle diameter (0.1–1, 1–1.8, 1.8–3, 3–6, and 6–10 μm)^21,22^. The scheme, based on the GOCART aerosol module, accounts for key characteristics of erodible surfaces, including particle size distribution, which determines the baseline erosion threshold and emission efficiency, and surface roughness, which modifies the effective friction velocity acting on the surface. In addition to these surface properties, soil moisture plays a critical role by increasing inter-particle cohesion and thereby raising the threshold friction velocity required for particle entrainment. When the actual friction velocity exceeds the threshold, particles are lifted into the atmosphere. The vertical dust flux is derived from the horizontal saltation flux following mass conservation principles, whereby the vertical flux is proportional to the horizontal flux divided by the characteristic particle travel distance, as described in^23,24^. Surface dust concentrations are subsequently simulated through atmospheric transport processes and constrained by the assimilation of aerosol optical depth (AOD) observations within the reanalysis framework^25^.

To the best of our knowledge, no studies have yet validated dust-related variables in the MERRA-2 reanalysis using observational data from Tehran, highlighting a significant gap and a promising avenue for future research^26^. reported a reasonable agreement between ERA5 and observed vertical wind shear values in Tehran. However, a key limitation of the present study is the lack of the spatial and temporal resolution required to fully capture diurnal-scale dust processes within the PBL^27^. As a result, the derived diurnal dust budgets should be interpreted with caution, recognizing that they do not fully account for sub-grid and fine-scale turbulent processes that significantly influence dust concentration. Furthermore, these datasets do not provide sufficient detail to capture the complex dust-PBL interactions. For instance, the surface cooling effect of dust directly restricts the daytime growth of the PBL. This ultimately leads to a shallower-than-expected PBL height^28,29^.

### Study area

**Fig. 1:**
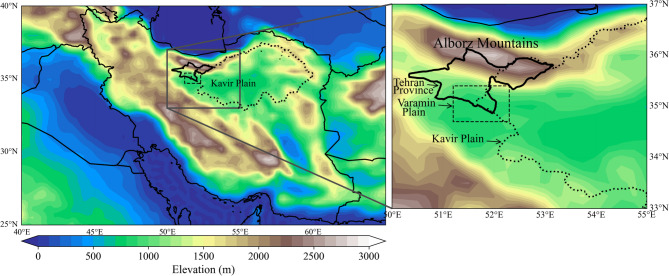
Geographic map of Iran showing the location of Tehran Province, the Varamin Plain, and the Kavir Plain. Regional topography is illustrated using color shading (meters above sea level), derived from ERA5 surface geopotential data.

Tehran Province is framed by the Alborz Mountains to the north and vast desert expanses to the south (Fig. 1). The region features dramatic elevation changes: the northern mountains rise to nearly 4,000 m above sea level, while the southern and southeastern areas, including the Varamin Plain (Fig. 1), dip to around 900 m, marking the lowest point in the province. The terrain gradually transitions from the low-lying plains in the south to the foothills at the southern edge of the Alborz Mountains, reaching elevations of about 1,800 m. To the southeast, the Kavir and Varamin Plains (Fig. 1) act as the primary sources of dust in Tehran Province, particularly during the summer months^6^.

### Methodology

To investigate the diurnal variability of dust-related quantities, we analyze the climatology of key dust variables, including dust emission flux, dust surface concentration, and dust horizontal flux, at different times of day during the summer months. We then assess the correlation between these variables and the factors driving their diurnal changes in Tehran Province by calculating Pearson correlation coefficients. Specifically, we calculate the Pearson correlation between dust surface concentration and PBL height, as well as between dust surface concentration and the zonal and meridional components of dust flux. In addition, we explore the correlation between dust emission flux and 10-m wind speed. Pearson correlation coefficients were calculated using the 24 long-term hourly mean values representing each hour of the diurnal cycle, enabling quantitative comparison of the shape and timing of their evolution.

For the dynamic analysis of diurnal variations in the vertical wind structure influencing dust transport, we utilize zonal and meridional wind components, vertical velocity, and the geopotential gradient from near-surface levels up to the 600 hPa level. Vertical profiles of vertical velocity and meridional wind components are analyzed to assess the thermal circulation induced by the Alborz Mountains, which influence the south-to-north wind direction. Additionally, the vertical profile of the zonal wind component is examined in conjunction with the vertical-meridional gradient of geopotential, which is also shaped by the presence of the Alborz Mountains. Evaluating these wind profiles provides valuable insights into the role of thermal circulation in driving horizontal dust flux and, ultimately, influencing dust surface concentration.

## Results

### Diurnal variation of dust emission and surface concentration

**Fig. 2:**
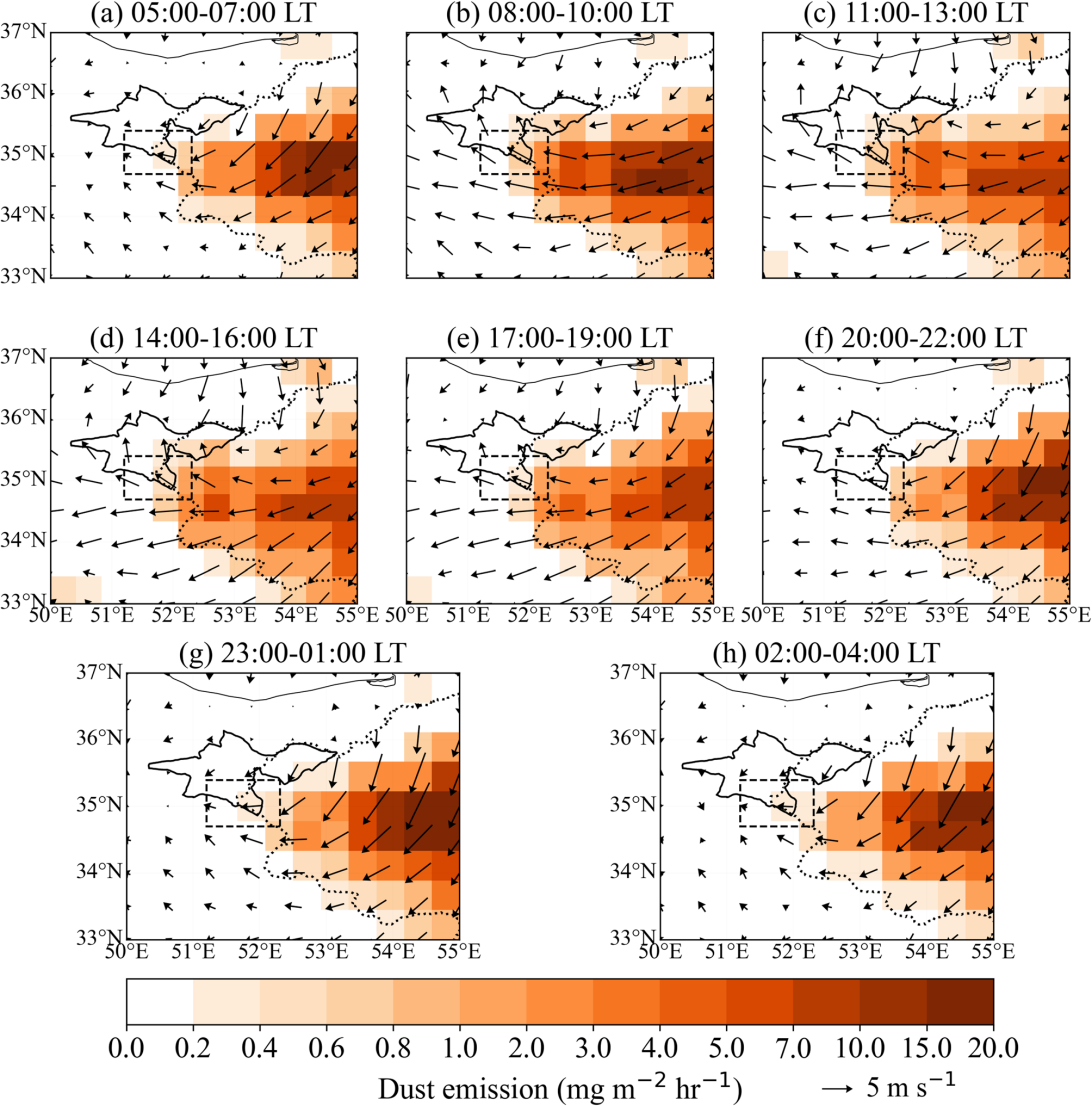
Mean dust emission flux (color shading, mg m⁻² h⁻¹) and 10-m wind (vectors, m s⁻¹) during the summer months (1980–2023) at different local times (LT). The boundaries of Tehran Province, the Varamin Plain, and the Kavir Plain are indicated by bold black, dashed, and dotted lines, respectively. Dust emission flux is derived from MERRA-2, and wind fields are obtained from ERA5.

Figure 2 illustrates the diurnal variability of dust emission flux and the 10-m wind vector during the summer months (1980–2023), based on MERRA-2 data. The figure shows that the primary source of dust emissions throughout the day is the Kavir Plain. Dust emission flux increases significantly in Tehran Province, particularly in the southern areas, between 08:00–10:00 and 11:00–13:00. During these periods, the dust emission flux in the Varamin Plain (Fig. 1) exceeds 5.2 and 4.2 mg m⁻² hr⁻¹, respectively. As southeastern upslope winds intensify, dust emissions (areas with flux greater than 0.4 mg m⁻² hr⁻¹) extend toward the central parts of Tehran Province (Figs. 2b–c). Simultaneously, the 10-m wind speed reaches its daily maximum in these regions.

Dust emission flux remains significant during 14:00–16:00 and 17:00–19:00 in the southeastern parts of Tehran Province. However, at night, as the downslope northerly and northeasterly winds develop, the 10-m wind speed decreases significantly (Figs. 2f–h). Between 02:00 and 04:00, dust emission flux drops to about 10% of the values observed between 08:00–10:00. Consequently, both the dust emission flux and 10-m wind speed in the southeastern parts of Tehran Province are much higher during the day than at night.

**Fig. 3:**
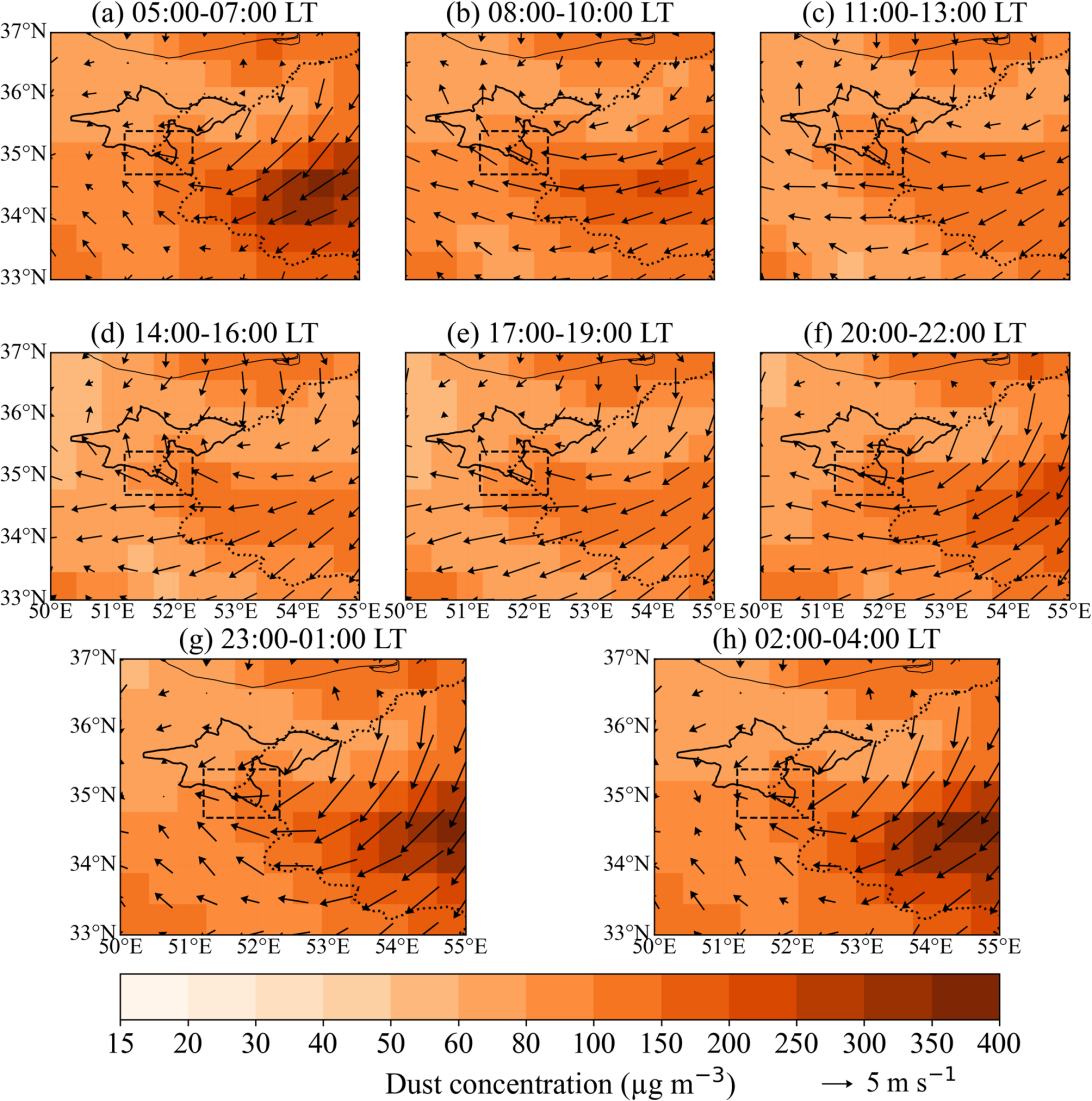
Similar to Fig. 2, but showing dust mass surface concentration (color shading, µg m⁻³) and 50-m wind (vectors, m s⁻¹).

Figure 3 shows that dust surface concentrations are highest over the Kavir Plain during the night and early morning (Fig. 3a, f–h). As the day progresses, the concentration over the Kavir Plain gradually decreases, while it first increases in the southern parts of Tehran Province and then in the northern areas. In the northern and central parts of Tehran Province, dust surface concentrations peak during the hours of 08:00–10:00 and 11:00–13:00 (Fig. 3b–c). Following this, between 14:00–16:00 and 17:00–19:00, concentrations decrease across the entire region (Fig. 3d–e).

As the day progresses, the southeasterly wind intensifies, leading to an increase in dust surface concentrations in Tehran Province and a decrease in concentration over the Kavir Plain and southern plains (Fig. 3b–c). During the hours of 08:00–10:00, with the increased dust emission in the southeastern parts of Tehran Province and the formation of southeasterly winds (Fig. 2b), dust surface concentrations first increase in the southern half, followed by an increase in the northern half of Tehran Province (Fig. 3b–c). In the northern parts, maximum dust surface concentrations occur between 11:00 and 13:00 (Fig. 3c). However, this peak may not be easily discernible in the figure, as dust concentrations are significantly higher over the Kavir and Varamin plains (Fig. 1) during both day and night.

**Fig. 4:**
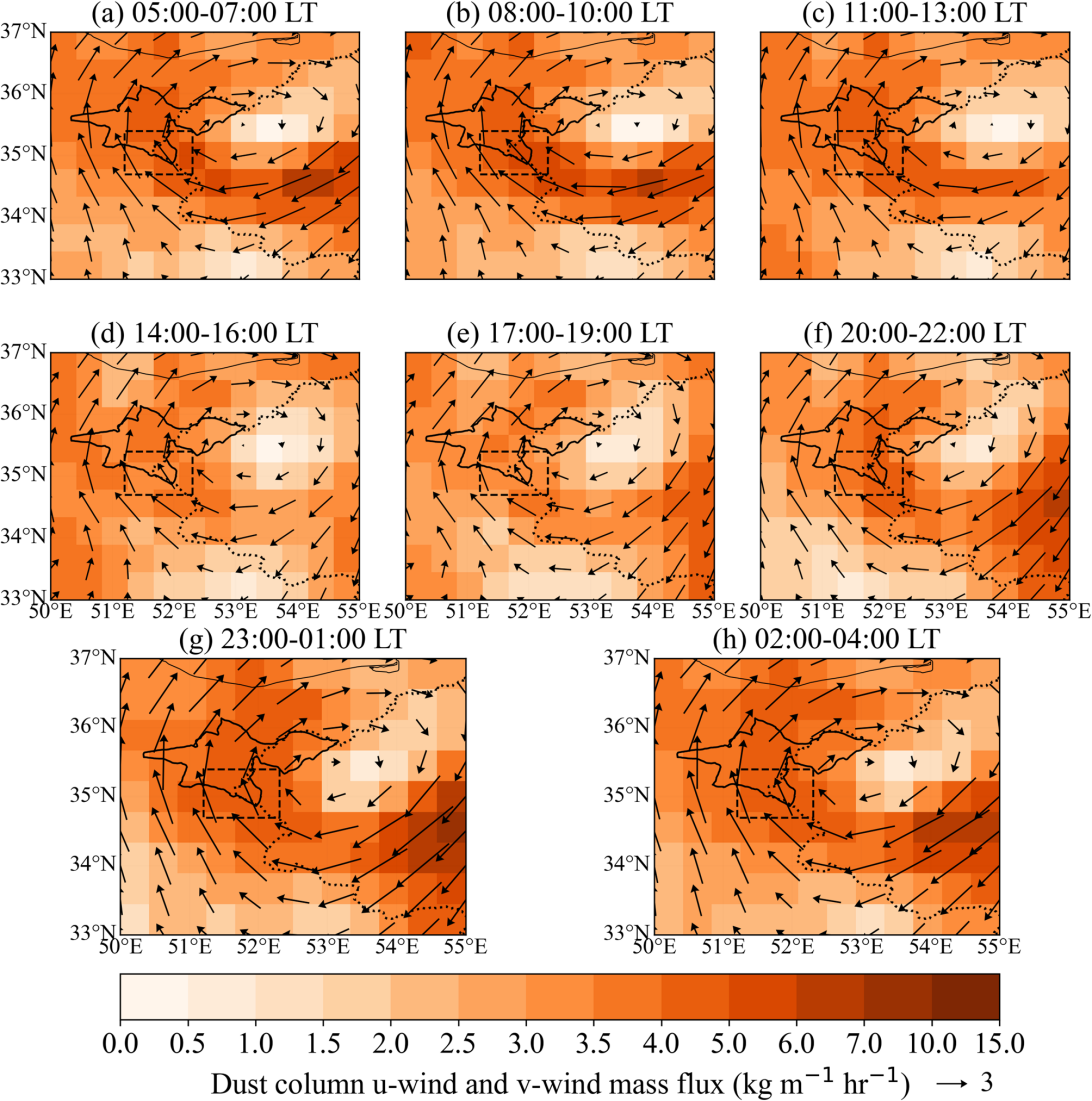
Similar to Fig. 2, but showing the magnitude (color shading, kg m⁻¹ hr⁻¹) and direction (vectors, kg m⁻¹ hr⁻¹) of dust column u-wind and v-wind mass flux.

### Diurnal variation of dust horizontal mass flux

Figure 4 shows the diurnal transport of dust from the southern plains to Tehran Province and the northern regions of the province during the summer. The analysis of the horizontal dust flux reveals that the highest flux toward Tehran Province occurs between 08:00 and 10:00, with the intensity being particularly high in the western part of Tehran Province (Fig. 4b). In the afternoon, however, the intensity of the horizontal dust flux decreases (Fig. 4d-e). As the dust surface concentration diminishes (Fig. 3d-e) and southeastern wind speed decreases (Fig. 2d-e), the horizontal dust flux reaches its minimum level of the day (Fig. 4d-e). During the night and early morning, the dust column u and v-wind mass flux increase again (Fig. 4a, f-h), corresponding with the rise in dust surface concentration (Fig. 3a, f-h).

### Observational evidence from surface PM2.5 and PM10 measurements

**Fig. 5:**
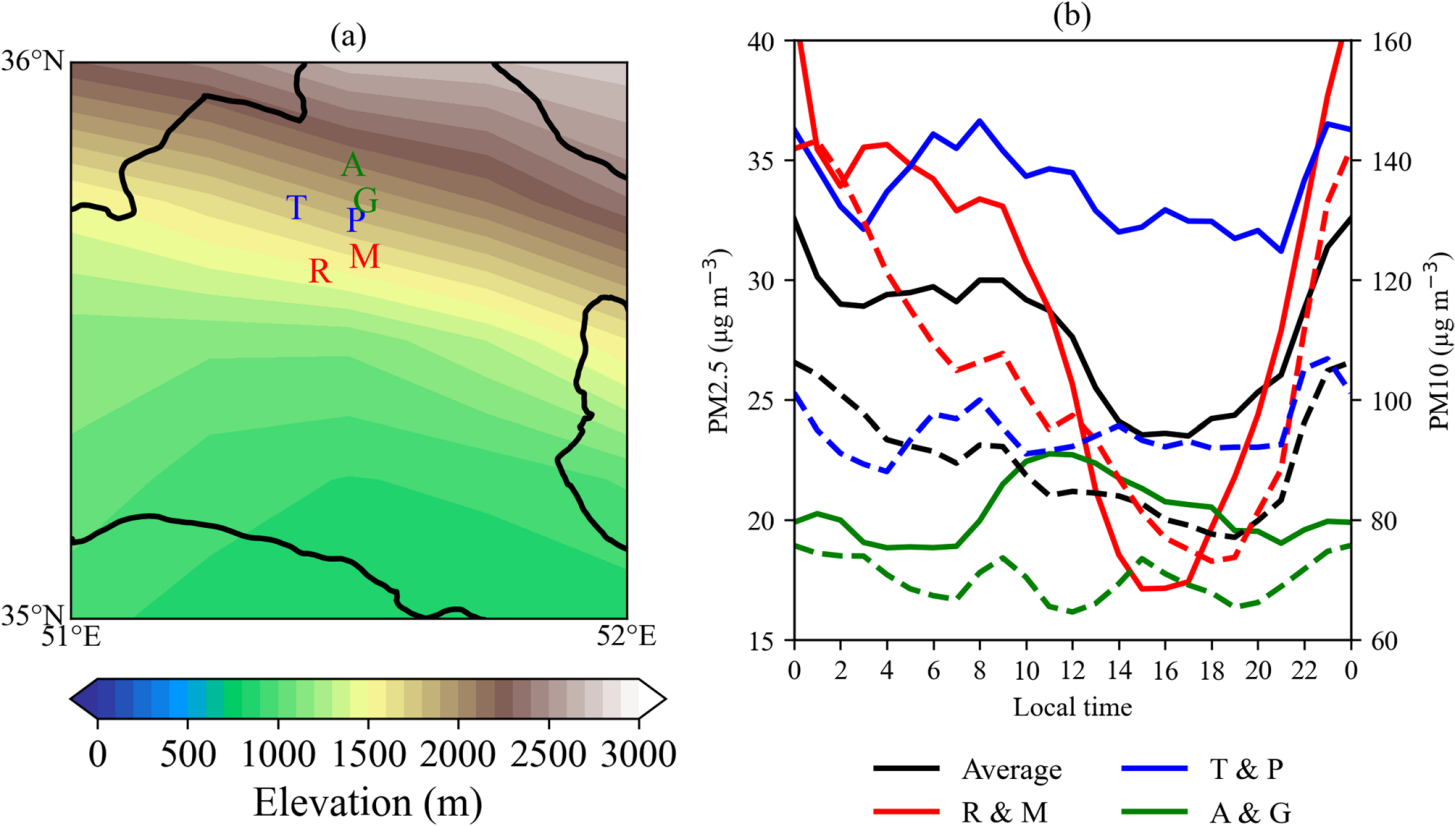
(**a**) Locations of the Air Quality Control Company monitoring stations used in this study, grouped into southern (Masoudieh, Rey), central (Piroozi, Tarbiat Modares), and northern (Aqdasiyeh, Golbarg) sectors of Tehran, overlaid on surrounding terrain elevation. (**b**) Mean diurnal variation of PM2.5 (solid lines, left axis) and PM10 (dashed lines, right axis) concentrations during summer (June–August) 2023, averaged over all stations (black) and separately for southern (red), central (blue), and northern (green) stations. In both panels, each station is represented by the first letter of its name.

To provide observational support for the reanalysis-based results, hourly PM2.5 and PM10 concentrations during summer 2023 were analyzed at six air-quality monitoring stations across Tehran, operated by the Air Quality Control Company (Fig. 5a). The stations were categorized according to their latitudinal position within the city and relative to the surrounding terrain: southern (Masoudieh, Rey), central (Piroozi, Tarbiat Modares), and northern (Aqdasiyeh, Golbarg).

As shown in Fig. 5b, the southern stations display a pronounced nocturnal increase in PM2.5 and PM10, with concentrations peaking around local midnight and remaining elevated until the early morning hours. A secondary peak occurs in the early morning, likely linked to locally elevated dust emission flux during those hours, combined with the strengthening of southeasterly low-level flow toward Tehran, a pattern also evident in the MERRA-2 dust emission and near-surface wind fields (Figs. 2, 3 and 4). The smaller amplitude of the morning peak compared to the midnight peak reflects the gradual transition toward daytime PBL development and increased vertical mixing.

During the daytime, PM2.5 and PM10 concentrations at the southern stations decline rapidly, reaching a minimum in the mid-to-late afternoon (around 16:00–18:00). This afternoon minimum coincides with the peak PBL height, which enhances vertical dilution of near-surface aerosols. In contrast, the afternoon decrease is markedly smaller at the central and northern stations (Fig. 5b). This spatial contrast indicates that continuous daytime dust transport toward the urban area and northern Tehran partially offsets the diluting effect of PBL deepening.

Distinct daytime PM2.5 and PM10 concentration peaks are observed at the central and northern stations, occurring around 08:00 LT for both PM2.5 and PM10 at the central stations, and around 12:00 (PM2.5) and 09:00 and 15:00 (PM10) at the northern stations. The progressive delay of the daytime maximum from south to north indicates a northward propagation of dust-laden air masses across the city, consistent with the diurnally evolving circulation and upslope transport inferred from the reanalysis fields. Overall, the observed variability in PM2.5 and PM10 concentrations aligns well with the MERRA-2–derived dust emission, transport, and concentration patterns shown in Figs. 2, 3 and 4, providing observational support for the proposed terrain-modulated, thermally driven dust transport mechanism.

### Diurnal correlations between dust and meteorological variables

**Fig. 6:**
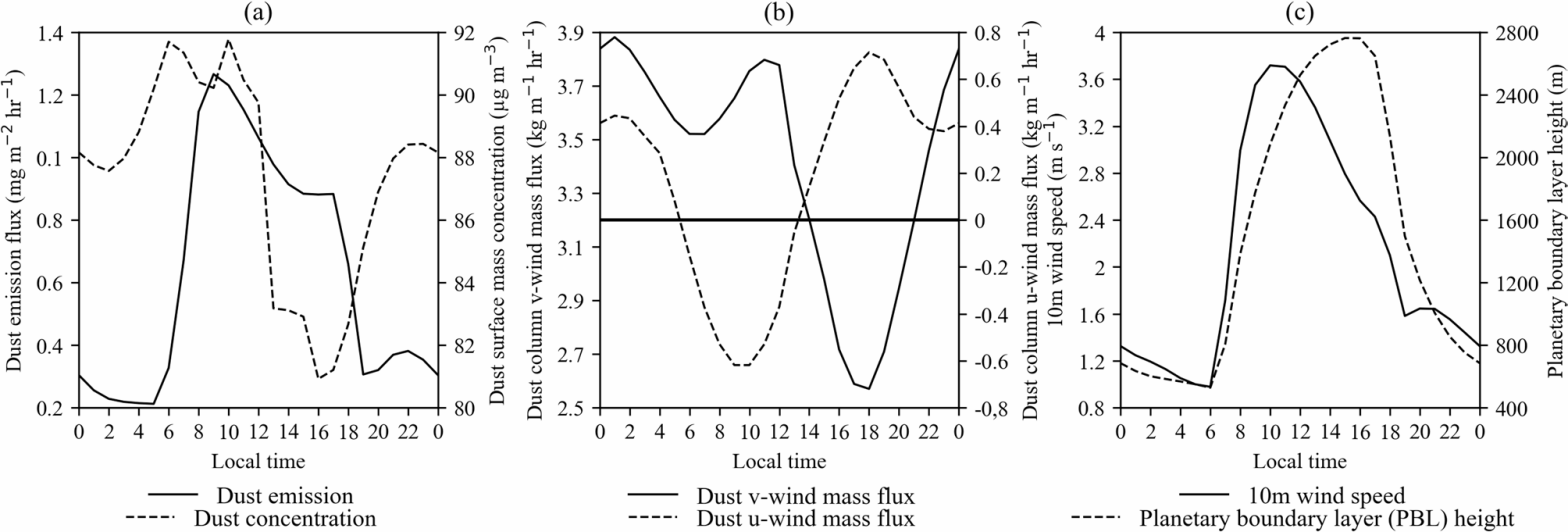
The average diurnal variations of dust-related quantities in Tehran Province (35°N to 36°N and 50.625°E to 51.875°E) during the summer months for the period 1980–2023, based on MERRA-2 data. (**a**) Dust emission flux (mg m⁻² hr⁻¹, solid line, left axis) and dust surface mass concentration (µg m⁻³, dashed line, right axis); (**b**) Dust column v-wind mass flux (kg m⁻¹ hr⁻¹, solid line, left axis) and dust column u-wind mass flux (kg m⁻¹ hr⁻¹, dashed line, right axis); (**c**) 10-m wind speed (m s⁻¹, solid line, left axis) and planetary boundary layer (PBL) height (m, dashed line, right axis) at different times of the day in local time (LT).

Figure 6 shows the diurnal variability of dust-related quantities, 10-m wind speed, and PBL height for Tehran Province. The highest dust emission flux in Tehran Province occurs at 09:00, reaching approximately 1.2 mg m⁻² hr⁻¹. This emission gradually decreases throughout the day, with a significant drop at night, falling to about 0.2 mg m⁻² hr⁻¹ (Fig. 6b). Dust surface concentrations increase after sunrise, peaking at around 92 µg m⁻³ at 10:00, before declining in the afternoon to a minimum of approximately 81 µg m⁻³ at 16:00 (Fig. 6c).

The diurnal variation of dust emission flux in Tehran Province is strongly correlated with near-surface wind speed, whereas variations in dust surface concentration are primarily associated with horizontal dust flux (Fig. 6). The correlation coefficient between dust emission flux and 10-m wind speed is 0.97, statistically significant at the 99% confidence level (Table 1). This strong association is physically consistent with the well-established dependence of dust emission on near-surface wind stress, whereby stronger winds enhance friction velocity and promote particle entrainment once the erosion threshold is exceeded^21,22^. Similarly, the correlation coefficients between dust surface concentration and the dust column u-wind and v-wind mass fluxes are approximately − 0.66 and 0.77, respectively, both significant at the 99% confidence level. The negative correlation with the dust column u-wind mass flux indicates that easterly flow is associated with increased dust surface concentration in Tehran Province during summer.

As shown in Fig. 6b, the highest dust surface concentration occurs when the dust column u-wind mass flux is easterly. Between 06:00 and 13:00, the zonal component of the dust flux is easterly, while it becomes westerly for the remainder of the day. During the easterly phase of the zonal dust flux, its maximum intensity occurs at 09:00 and 10:00, reaching 0.61 kg m⁻¹ hr⁻¹ (Fig. 6b), which coincides with the peak dust emission and dust surface concentration (Fig. 6a). When the dust column u-wind mass flux is westerly, its maximum intensity occurs at 18:00, with a value of 0.71 kg m⁻¹ hr⁻¹ (Fig. 6b).

Despite changes in the direction of the zonal component of the dust flux, the meridional component remains positive throughout the day and night, indicating the transport of dust from the southern regions to the northern part of Tehran Province. The meridional component of the dust flux is approximately five times larger than the zonal flux (Fig. 6b). Furthermore, the dust column v-wind mass flux exhibits two peaks, occurring at 02:00 and 11:00, with values of 3.83 and 3.79 kg m⁻¹ hr⁻¹, respectively. Analysis of both the meridional and zonal components suggests that from 06:00 to 13:00, dust is transported into Tehran Province from the southeast, whereas during the remaining hours, transport occurs from the southwest (Fig. 6b).

### Diurnal variability of dust processes driven by PBL height and wind patterns

In Tehran Province, particularly in the northern regions, the dust emission flux is minimal during nighttime hours (Fig. 2f–h). Additionally, a gentle, thermally-driven northerly mountain breeze acts as a barrier, limiting the northward transport of dust emitted from the southern plains. Together, these two factors help counteract the expected increase in dust surface concentration that would typically result from the reduced PBL height during the night in these areas.

As the 10-m (Fig. 2b–c) and 50-m (Fig. 3b–c) southeasterly winds strengthen from early morning to midday, the transport of dust from the southern plains toward the Alborz Mountain slopes intensifies (Fig. 4a, c). In the afternoon, these southeasterly winds weaken, dust emissions decline (Fig. 2d–e), and the PBL height reaches its peak (Fig. 6c), resulting in the lowest surface dust concentrations of the day (Figs. 4d–e and 6c).

The temporal and spatial distribution of dust concentration is shaped by variations in dust emission flux, PBL height, and wind speed and direction. At night, while dust emission in the Kavir and Varamin plains remains high (Fig. 2f–h), it declines sharply compared to daytime levels (Fig. 2b, d). Despite the reduction in 10-m wind speed during nighttime hours (Fig. 2f–h), an intensifying low-level jet enhances momentum transfer to the surface, triggering dust emissions in the plains^30^. Unlike northern Tehran, the Kavir and southern plains are less affected by gentle downslope winds. Additionally, the reduction in PBL height during the night and early morning (Fig. 6c) contributes to increased surface dust concentrations^12^. As a result, peak dust concentrations occur in the Kavir and Varamin plains during these hours (Fig. 3a, f–h).

It is important to note that the dust emission flux observed in Tehran Province (Fig. 6a) primarily originates from the Varamin Plain and adjacent southern areas, which constitute the main dust sources of the region. The central and northern parts of the province contribute negligibly to dust emissions, so the diurnal pattern in Fig. 6a closely mirrors that of the Varamin Plain, albeit at a lower magnitude. While dust advection from these sources can influence surface dust concentrations in Tehran, this relationship is strongly modulated by local PBL height. According to Fig. 6c, the PBL reaches its maximum height of approximately 2760 m at 15:00–16:00, coinciding with the lowest dust surface concentration, whereas the minimum PBL height occurs at 06:00, dropping to less than 25% of its maximum value. However, due to the combined influence of horizontal flux, dust emission flux, and wind speed and direction, the minimum PBL height does not correspond exactly to the maximum surface dust concentration. The correlation coefficient between dust surface concentration and PBL height is − 0.59, statistically significant at the 99% confidence level (Table 1). This negative correlation is strongest over the southern areas and gradually weakens toward central and northern Tehran, where enhanced daytime dust transport reduces the correspondence between minimum PBL height and maximum surface dust concentration. Consequently, peak dust concentrations do not always coincide with the lowest PBL heights, indicating that boundary-layer dynamics do not uniformly control dust variability across all subregions.

It should be noted that the spatial averaging applied in Fig. 6 introduces some uncertainty, as it represents averages over four MERRA-2 grid boxes encompassing both lowland desert and mountainous terrain, limiting the ability to fully separate plain and mountain influences. Furthermore, dust column and u- and v-component wind mass fluxes are interpreted here as indicators of the overall atmospheric transport pathway rather than solely near-surface transport. To address these limitations, Table 1 provides a quantitative reference for the relationships among the variables, complementing the discussion of their diurnal evolution.

**Table 1:** Pearson correlation coefficients between area-averaged dust-related and meteorological variables over Tehran Province (35°–36°N, 50.625°–51.875°E), computed from 24 long-term hourly climatological means during the summer months (1980–2023). Asterisk indicates correlations significant at *p* < 0.01.

	Dust emission flux	Dust surface mass concentration	PBL height	Dust column v-wind mass flux	Dust column u-wind mass flux	10-m wind speed
Dust emission flux		-0.06	0.78*	-0.06	-0.65*	0.96*
Dust surface mass concentration	-0.06		-0.59*	0.77*	-0.66*	-0.10
PBL heaight	0.78*	-0.59*		-0.49	-0.13	0.83*
Dust column v-wind mass flux	-0.06	0.77*	0.49		-0.55*	-0.04
Dust column u-wind mass flux	-0.65*	-0.66*	-0.13	-0.55*		-0.60*
10-m wind speed	0.96*	-0.10	0.83*	-0.04	0.60*	

### Diurnal wind circulation induced by the Alborz Mountains

**Fig. 7:**
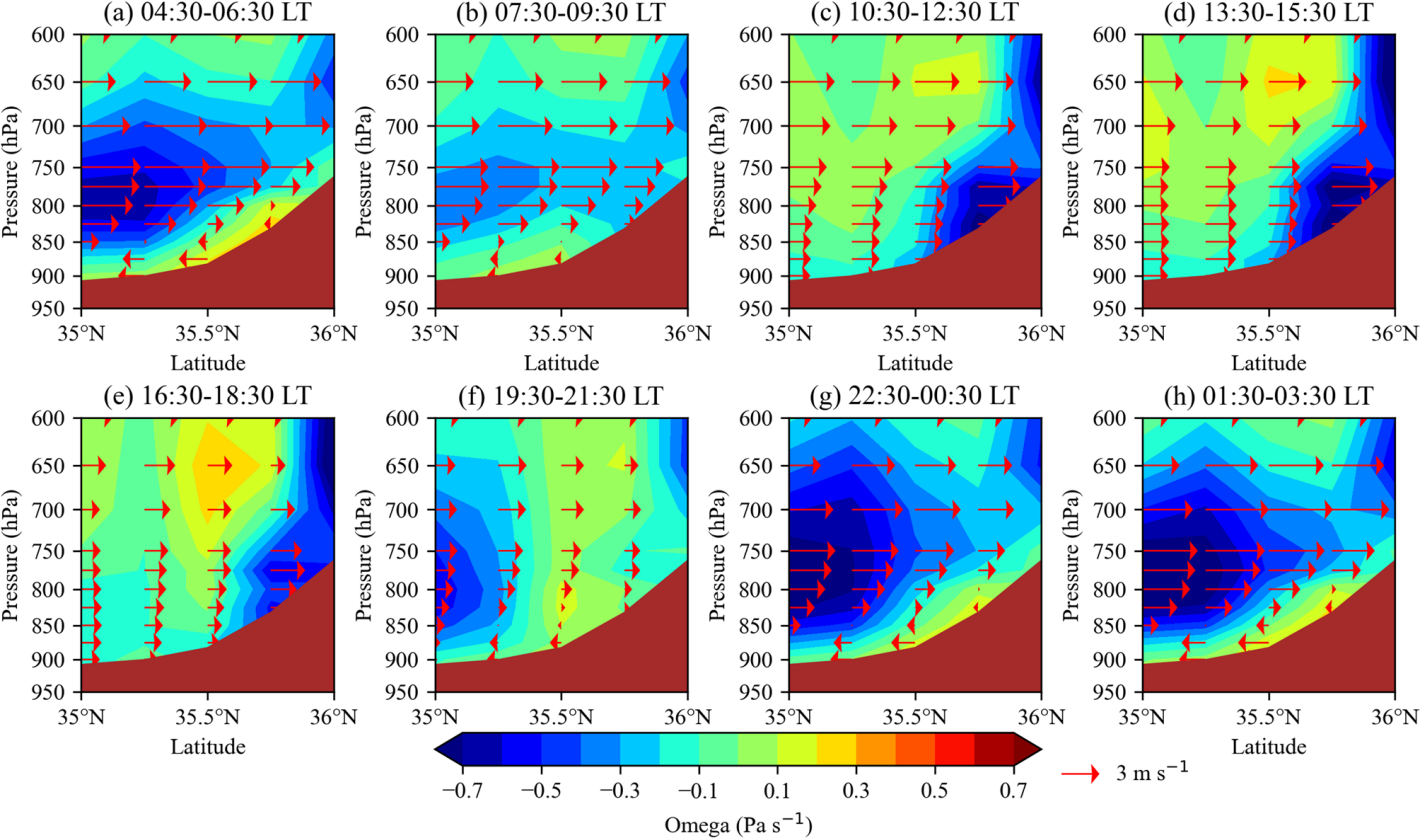
The average meridional-vertical cross-section of vertical velocity (color shading, Pa s⁻¹), along with the meridional wind component (vectors, m s⁻¹), at a longitude of 51.25°E during the summer months (June, July, and August) of 2023, at different times of the day in local time (LT), based on ERA5 data. The brown color represents the Alborz Mountains and their corresponding elevations.

Figure 7 illustrates the influence of the Alborz Mountains and their slopes on changes in the meridional wind component, which plays a critical role in dust emission, transport, and surface concentration in Tehran Province during summer. During the day, shortwave solar radiation absorbed by the surface leads to higher air temperatures in the boundary layer over the mountain slopes compared to the adjacent plains. This thermal contrast creates a horizontal pressure gradient, with relatively lower pressure over the heated slopes and higher pressure over the cooler plains. As a result, upslope winds develop, and air ascends over the Alborz Mountains. To compensate for the rising air, air from the southern plains flows northward, reinforcing the southerly wind component. The greater the temperature difference between the mountains and the plains, the stronger this upslope circulation becomes. Consequently, during midday (10:30–15:30), when solar heating is strongest, near-surface southerly winds reach their peak intensity (Fig. 7c–d).

At night, the air temperature near the surface over the mountains becomes slightly cooler than that over the plains at the same pressure level. As a result, during the night and early morning (22:30–06:30), the denser air over the mountains creates a light breeze near the surface, flowing from the Alborz Mountain slopes towards southern parts of Tehran Province (Fig. 7a, f–h). The transition between the downslope northerly winds and the upslope southerly winds occurs between 07:30–09:30 and 16:30–18:30, respectively (Fig. 7b, e). Despite the diurnal shift in meridional wind components at lower altitudes in Tehran Province, the meridional wind component above 800 hPa remains consistently southerly (Fig. 7). Unlike the northerly wind near the surface, the southerly wind above 800 hPa is stronger at night than during the day.

The diurnal variations in the meridional component of the dust flux help explain some of the changes in dust surface concentration. The meridional dust flux exhibits two peaks during the day. The first peak, between 23:00 and 03:00 (Fig. 6b), coincides with the maximum southerly wind speed at 600–800 hPa (Fig. 7a, g-h). During this time window, a strong upward motion is also observed between 35.0° and 35.5°N at 850–650 hPa (Fig. 7g–h), likely resulting from thermally-induced upslope flow over the Alborz mountain slopes. This upward motion appears to be part of a localized mountain–plain circulation, rather than being driven by large-scale upper-level westerlies. The second peak occurs between 10:00 and 12:00 (Fig. 6b), corresponding to the maximum southerly wind speed at 800–900 hPa (Fig. 7c–d). Therefore, during the first peak, dust transport to Tehran Province primarily originates from altitudes above 800 hPa. In contrast, during the second peak, dust is transported from both higher altitudes and near the surface, leading to the highest dust surface concentration in Tehran Province during the late morning hours (Fig. 6b).

It should be noted that the spatial resolution of ERA5 is insufficient to fully resolve fine-scale mountain–valley and slope wind circulations over the rugged Alborz Mountains. As a result, sub-grid topographic effects may locally influence wind strength and timing. Nevertheless, ERA5 is able to capture the larger-scale diurnal wind reversals and pressure-gradient-driven circulations that control basin-scale dust transport, which are the primary focus of this study.

### Diurnal variation of zonal wind and geopotential gradient

**Fig. 8:**
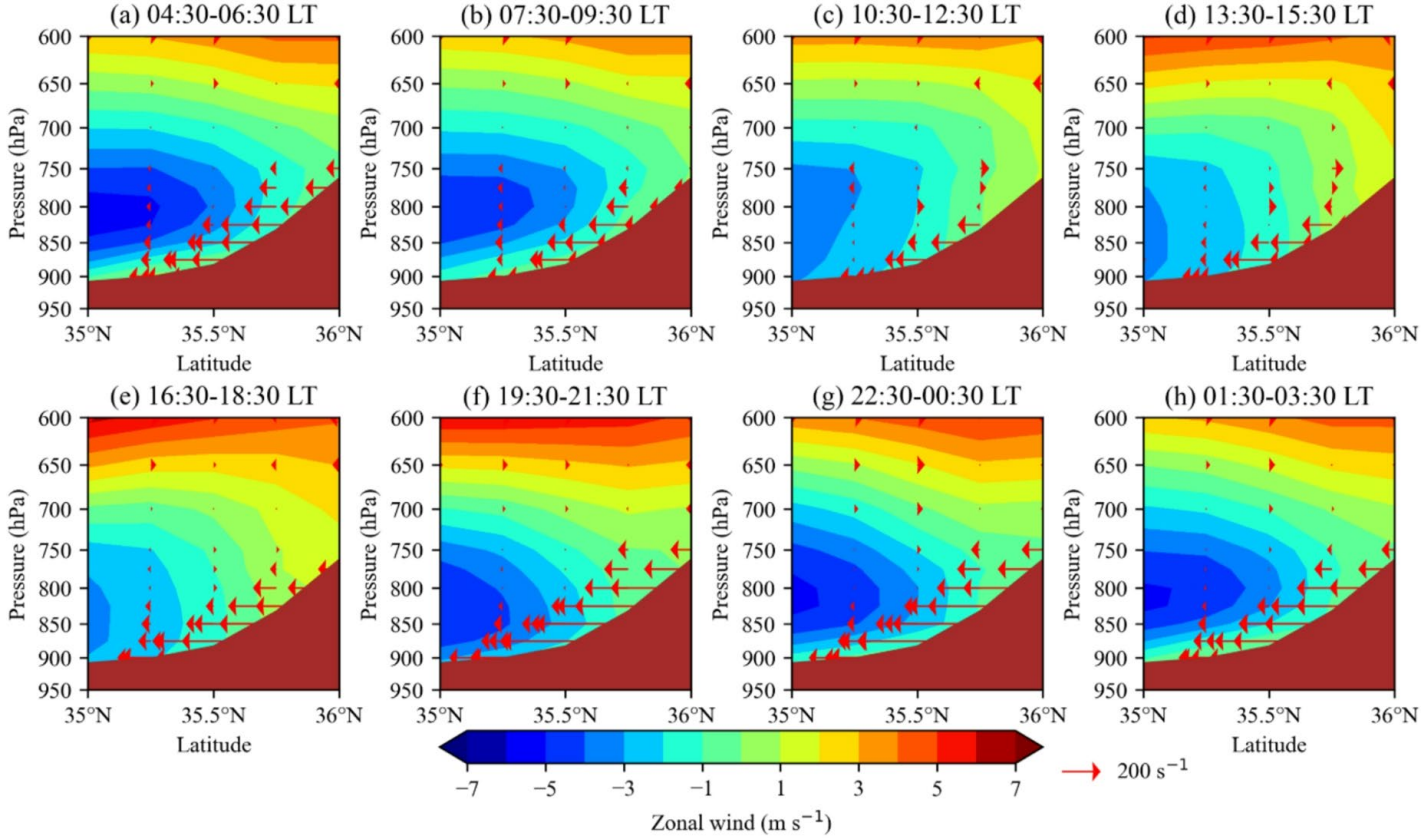
The average meridional-vertical cross-section of the zonal wind component (color shading, m s⁻¹), along with the meridional geopotential gradient (vectors, s⁻¹), at a longitude of 51.25°E during the summer months (June, July, and August) of 2023, at different times of the day in local time (LT), based on ERA5 data. The brown color indicates the Alborz Mountains and their corresponding elevations. The direction of the geopotential gradient vectors is from high to low.

In Tehran Province, the average wind direction in the vertical atmospheric column is westerly. However, at lower atmospheric levels during the summer, the dominant wind direction is easterly, as indicated in Figs. 2 and 3. This easterly wind is driven by the high-pressure system over the Alborz Mountains and the low-pressure system over the Kavir Plain and the southern plains of Tehran Province (data not shown). Its intensity exhibits diurnal variation, as shown in Fig. 8. Over the plains of Tehran Province, the easterly wind prevails between 850 hPa and approximately 700 hPa, with its intensity increasing at night due to a stronger meridional geopotential gradient (Fig. 8). This vertical wind profile and its nocturnal intensification are consistent with radiosonde observations from Mehrabad airport synoptic station at 00:00 and 12:00 local time (not shown).

At night and in the early morning (22:30–06:30), radiative cooling causes surface pressure to increase and atmospheric thickness to decrease over the mountains. This results in a stronger meridional geopotential gradient in the lower atmosphere, which intensifies the easterly winds around 800 hPa (Fig. 8a, g-h). During the day, while the easterly wind at 800 hPa weakens, the intensity near the surface increases (Fig. 8c-e). This is due to increased turbulent mixing, which enhances vertical momentum transfer^31^ and allows the easterly wind from 850 to 700 hPa to reach the surface with greater intensity. The maximum easterly wind speed near the surface occurs between 10:30 and 12:30, reaching about 4 m s⁻¹ at 900 hPa at 35°N (Fig. 8c). As a result, the peak in easterly wind speed at 900 hPa (Fig. 8c) coincides with the maximum easterly dust mass flux, which occurs between 09:00 and 10:00 (Fig. 6b).

Between 06:00 and 13:00, the strengthening southeasterly flow enhances dust transport from the Varamin and Kavir plains. After 13:00, as this flow weakens, surface dust transport from the Kavir Plain diminishes, leading to a net westerly dust mass flux (Fig. 6b). This shift is accompanied by increased dust transport from the western regions at higher altitudes, as westerly winds intensify in the afternoon. Additionally, Fig. 8e shows that at mid-levels of the atmosphere (600–650 hPa), westerly wind intensity increases in the afternoon, peaking between 16:30 and 18:30, coinciding with the maximum westerly dust mass flux at 18:00 (Fig. 6b). During the transition between day and night, specifically from 07:30 to 09:30 and from 19:30 to 21:30, there is a downward shift in the easterly wind in the morning and an upward shift in the evening (Fig. 8b, f).

In summary, both the meridional and zonal wind components at pressure levels near the Earth’s surface (e.g., the 900 hPa level) reach their maximum values in Tehran Province around midday (10:30–12:30). This coincides with the strongest dust horizontal mass flux and, consequently, the highest dust surface concentration. The increase in wind speed from morning to midday is likely due to enhanced momentum transfer from higher atmospheric levels to the surface, which peaks around noon (approximately at 11:00) (Fig. 6c).

## Discussion

In this study, we used hourly MERRA-2 data for 1980–2023 during the summer months to examine long-term diurnal variations of dust-related variables, and ERA5 data for summer 2023 to analyze wind speed and direction at various pressure levels, which are critical for dust emission and transport. In addition, hourly surface PM2.5 and PM10 measurements from six air-quality monitoring stations in summer 2023 were analyzed to provide observational support. MERRA-2 provided insights into dust variables, while ERA5 and the surface observations allowed evaluation of wind-driven transport and near-surface dust patterns.

Our results indicate that surface topography, through its influence on wind fields, strongly shapes the spatial and temporal distribution of summer dust concentrations. At southern stations, both PM2.5 and PM10 exhibit pronounced nocturnal peaks around midnight, remaining elevated until early morning, followed by a secondary morning peak associated with enhanced local dust emission and southeasterly low-level flow. Dust surface concentration is generally inversely related to PBL height, which is lowest at night and early morning and peaks in the afternoon. In central and northern stations, however, daytime maxima are observed, with PM2.5 peaking around 08:00 and PM10 showing multiple peaks between 09:00 and 15:00, while nocturnal concentrations are comparatively low. The difference between nighttime and daytime PM10 concentrations is substantially smaller in central and northern areas than in the southern plains (Fig. 5b), indicating that daytime dust inflow partially offsets the dilution associated with the rising PBL.

Together, these patterns suggest that northward transport of dust-laden air, modulated by terrain and thermally driven circulations, governs the observed spatial and temporal variability across Tehran. Similar diurnal variations have been reported in other complex terrains. For example, at the Zhangye site in northwestern China, trace gas concentrations decrease during the daytime due to enhanced vertical mixing and desert-origin winds, while PM10 and light-scattering coefficients exhibit pronounced morning and afternoon peaks associated with dust advection and local emissions^32^. These findings underscore the broader relevance of our results, suggesting that the interplay among boundary-layer growth, local dust emission, and regional transport driven by thermally induced circulations represents a common mechanism controlling diurnal dust variability in arid and semi-arid regions with complex terrain.

Early morning upslope winds develop and reach maximum intensity around midday, transporting dust from southeastern source regions first to central Tehran and then to northern areas. This advective flux contributes to elevated daytime concentrations in the central and northern regions despite increasing PBL height, explaining the weaker correlation between dust surface concentration and PBL in these areas compared to the southern plains, consistent with previous findings^33^. Overall, dust surface concentrations are higher in southern Tehran, aligning with observations of suspended particles reported by^7^.

The timing of surface dust peaks also reflects transient imbalances between advective influx and vertical dilution. In southern stations, the afternoon PBL growth exerts a strong controlling influence, leading to pronounced daytime decreases in PM2.5 and PM10. In central and northern stations, sustained dust transport moderates these decreases, producing smaller day–night contrasts, as supported by both reanalysis and observational studies. These patterns are consistent with previous reports of early-morning PM2.5 and PM10 peaks in Tehran^7,10^, while our analysis also identifies secondary daytime peaks in central and northern areas driven by advective dust transport.

Zonal and meridional wind components at multiple atmospheric levels play a key role in shaping dust flux and transport. The zonal component of dust mass flux shifts from easterly between 06:00 and 13:00 to westerly at other times, linked to the nocturnal easterly flow at 800 hPa descending toward the surface during the morning via enhanced vertical momentum transfer and breakdown of the low-level jet^31^. Comparison with dust column u- and v-wind fluxes indicates that dust transport to Tehran occurs from the southeastern regions near the surface in the early morning to midday and from southwestern regions primarily above 800 hPa for the remainder of the day.

**Fig. 9:**
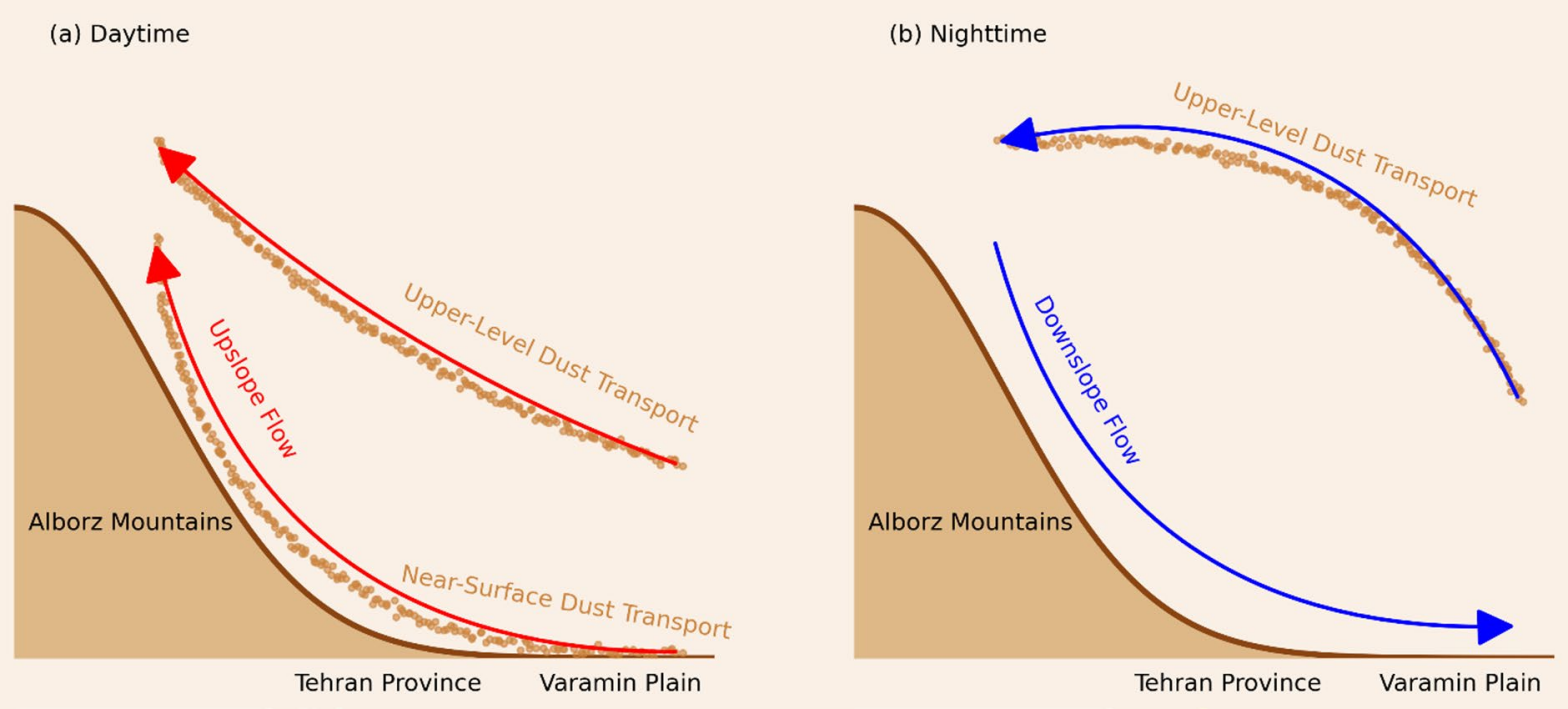
Schematic illustration of dust transport mechanisms in Tehran Province, derived from the analysis of horizontal dust flux and wind profile data.

Figure 9 summarizes these dynamics. During the day, upslope winds at both near-surface and upper levels carry dust toward the Alborz Mountains, while nighttime downslope flows confine near-surface transport, leaving dust movement primarily aloft under background synoptic flow. Unlike dust surface concentrations, which exhibit complex spatial patterns, diurnal variations in dust emission flux closely follow 10-m wind speed, peaking from early morning to midday and consistent with observations from the North African desert^34^. Morning increases in wind speed are driven by vertical atmospheric coupling induced by surface heating, which breaks down the nocturnal low-level jet and enhances dust emission^31^. Nighttime dust emission declines under gentle downslope winds from the northeast.

While MERRA-2 and ERA5 provide valuable insights into long-term dust dynamics, their coarse horizontal resolution limits the capture of fine-scale topographical and meteorological features critical to dust processes in Tehran Province. This may introduce uncertainty in correlations between dust concentrations and meteorological variables. Additionally, the lack of dense ground-based observational validation restricts certainty in the findings. Future studies should integrate advanced measurement techniques, such as wind and aerosol lidar systems, alongside surface dust concentration monitoring to improve understanding and prediction of dust events in this complex terrain.

## Conclusions

This study highlights the significant influence of thermally-driven flows induced by Tehran Province’s complex topography on diurnal variations in dust emission flux and surface dust concentration during the summer months. Dust transport patterns exhibit clear diurnal shifts driven by upslope winds during the day and downslope flows at night, with transport pathways varying vertically and spatially across the region.

The schematic presented in Fig. 9 encapsulates these dynamics, illustrating how diurnal cycles of topographically forced winds regulate dust emission and movement, shaping observed spatial and temporal dust patterns. The inverse relationship between dust concentration and PBL height, alongside the strong correlation between dust emission and near-surface wind speed, underscores the importance of coupling meteorological and topographical processes in understanding dust variability.

Although the study advances understanding of dust dynamics in Tehran Province, limitations related to the resolution of reanalysis data and lack of in situ validation highlight the need for future observational campaigns. Incorporating vertical profiles of wind and aerosol concentration, combined with surface measurements, will be crucial for refining dust event prediction and mitigation strategies.

Improved insights into diurnal dust processes will support more accurate hourly dust forecasts, which are vital for addressing air quality, visibility, and health risks posed by dust storms in Tehran Province and similar complex terrain regions worldwide.

## Data Availability

The MERRA-2 and ERA5 datasets are publicly available and can be accessed through the following links: MERRA-2: https://disc.gsfc.nasa.gov/datasets?project=MERRA-2, ERA5: https://cds.climate.copernicus.eu/datasets/reanalysis-era5-pressure-levels-monthly-means?tab=overview.
